# High prevalence of intestinal worms in children up to 5 years of age in Huaphan province, Lao People's Democratic Republic (PDR)

**DOI:** 10.1016/j.parepi.2017.06.001

**Published:** 2017-06-16

**Authors:** Naphavanh Nanthavong, Antony P. Black, Phonepadith Khattignavong, Lavy Lorphachan, Keooudomphone Vilivong, Sylvie Goossens, Yves Buisson, Fabrice Quet, Claude P. Muller, Satoshi Nakamura

**Affiliations:** a Institut de la Francophonie pour la Médecine Tropicale, Vientiane, Lao PDR; b Institut Pasteur du Laos, Vientiane, Lao PDR; c Independent Consultant, Vientiane, Lao PDR; d Luxembourg Institute of Health, Luxembourg, Luxembourg; e National Center for Global Health and Medicine, Japan; f Hiroshima Bunka Gakuen University, Hiroshima, Japan

**Keywords:** Lao PDR, Soil-transmitted-helminths, Prevalence

## Abstract

**Background:**

The prevalence of soil-transmitted helminth infection is high in Lao People's Democratic Republic (PDR), reaching 62% among school-children. However, this prevalence presents wide regional variations, due to differences in healthcare access and environmental factors. Curiously, there are few studies on helminth infections in pre-school children – an age group targeted by the national de-worming campaign. Therefore, a preliminary study was conducted in a remote region of Huaphan Province, North Laos, to determine the prevalence of helminth infections in pre-school children.

**Results:**

A total of 74 pre-school aged children provided stool samples for this study. Parasite eggs were detected in 41.9% with *Ascaris lumbricoides* being most common (32.4%). Presence of parasites was significantly associated with distance from health centres.

**Conclusions:**

Such a high prevalence of helminth infection indicates that the national deworming campaign is not adequate in the remote areas, in particular in villages distant from health care centres. It is necessary to ensure the proper administration of anti-helminthics to all children and to adapt the implementation of deworming campaigns to the specificities of each province.

## 1 Introduction

Due to easier accessibility and higher perceived burden of infection, the majority of studies on intestinal parasitic infections in Lao PDR have focused on adults or school-aged children (Sayasone et al., 2009, Rim et al., 2003, Watthanakulpanich et al., 2013, Conlan et al., 2012, Soukhathammavong et al., 2012), with scarce data on preschool-aged children (Sayasone et al., 2011, Kounnavong et al., 2011). For example, a nationwide study looking at prevalence of intestinal parasite infections among primary school children between 2000 and 2003 indicated high prevalence of helminth infection (61.9%) with large regional variation (Rim et al., 2003). This and other studies stimulated the introduction of a national deworming programme, which was initiated at the school level in 2005 (National Health Statistic Report FY 2010–2011, 2011). However, pre-school children are at high risk of infection and it has been argued that, in order to reduce malnutrition, deworming programmes should be implemented at an earlier age (Davis et al., 2014, Victora et al., 2010). Thus, the national deworming programme in Lao PDR was extended to pre-school aged children in 2006 (National Health Statistic Report FY 2010–2011, 2011). Whilst a number of authors have suggested that the deworming programme is a success, as indicated by high rates of drug delivery (Phommasack et al., 2008, Jex et al., 2011), others have stated that this conclusion is premature and that a more robust monitoring is required to assess the impact (Conlan et al., 2012).

In this study, we aimed to determine the parasite profile and prevalence among pre-school children aged 1–5 years in Xam Tai and Kuan districts, Huaphan province.

## 2 Methods

The investigation was part of a larger vaccination study carried out between May and July 2013, in which 26 villages (14 in the district of Xamtai and 12 in the district of Kuan) were randomly selected (Nanthavong et al., 2015). Out of the 132 parents involved in the larger study, 74 consented for their children to provide additional stool samples. Parasite infection was determined by formalin-detergent concentration and microscopy (Waikagul et al., 1977). Nutritional status was determined by Mid-Upper Arm Circumference (MUAC) and weight for height, height for age and weight for age z-scores. Data were collected on ethnicity, distance from the nearest health centres and vaccination status.

## 3 Results and discussion

The presence of parasite infection was found to be high in our cohort. Out of 74 stool samples, 31 (41.9%) had detectable eggs (Table 1). Another study in a similar age group in the south of Lao PDR found a similar prevalence of 37.7% (Kounnavong et al., 2011). This prevalence is lower than those reported in school aged children prior to the deworming strategy (91.2% in Huaphan) (Rim et al., 2003), this may be due to a beneficial effect of the deworming campaign. Nevertheless a lower exposure within our younger cohort may also partially contribute to this difference. Indeed, in our cohort there was a significant difference in the ages of children with parasites (mean age 43.7 months) as compared to those without (36.9 months, p = 0.01).

**Table 1:** Helminth eggs found in the faeces of children from Huaphan.

	Total (%)
*Ascaris lumbricoides* only	12 (16.2)
*Ascaris lumbricoides*/*hookworm*	3 (4.1)
*Ascaris lumbricoides*/*Trichuris trichiura*	4 (5.4)
*Ascaris lumbricoides*/*Trichostrongylus*	4 (5.4)
*Ascaris lumbricoides*/*Trichostrongylus*/*Trichuris trichiura*	1 (1.4)
*Enterobius vermicularis* only	1 (1.4)
*Hookworm only*	4 (5.4)
*Trichuris trichiura* only	2 (2.7)
Any parasite detected	31 (41.9)

The soil-transmitted helminth, *Ascaris lumbricoides*, was the most prevalent parasite, detected (alone or in mixed infections) in 21 of the samples (28.4%). A similar prevalence of *Ascaris* infection (27.4%) was found in pre-school aged children in the south of Lao PDR (Kounnavong et al., 2011), but this rate is higher than reported in Huaphan in 2009 by Conlan et al. (18.6%) (Conlan et al., 2012) among an older age group. However, as the authors themselves point out, there is considerable spatial, ethnic and wealth related variation in parasite prevalence, even within the same province.

Other parasite eggs detected in our study were *Trichuris trichiura* (whipworm; 9.5%), *Trichostrongylus* (roundworms; 6.8%) (Watthanakulpanich et al., 2013), hookworm; 9.5% and *Enterobius vermicularis* (pinworm; 1.4%). These infections are especially common in areas with poor sanitary conditions. Importantly, 12 individuals (16.2%) had mixed infections – believed to have a more detrimental effect on aspects such as cognitive performance than single infections (Jardim-Botelho et al., 2008).

All of the children in our study were less than 5 years of age and therefore were targeted by the pre-school deworming programme. This consists of Mebendazole, given twice per year. Although Mebendazole has been shown to have low efficacy against *Trichuris trichiura*, which was detected in 7 of our samples (9.5%), it has high efficacy for *A. lumbricoides* in the Lao population (Soukhathammavong et al., 2012). The local healthcare workers, operating out of the district health centres, usually administer Mebendazole during outreach services. Although nationwide coverage is reported to be high (93.2% of targeted preschool children in 2011 (National Health Statistic Report FY 2010–2011, 2011)), it remains possible that drug coverage in the district studied here was suboptimal. Indeed, vaccine coverage in the same area, which also depends on outreach from the local health centre, is low. Using vaccination records or parental recall, when no records were available, it was determined that only 59.8% of 132 children had received all 3 diptheria-tetanus-pertussis containing vaccine. Importantly, we found that individuals with parasites were living significantly further from healthcare centres than those without (mean distances of 48.7 km and 36.3 km, respectively; p = 0.02). Of note, a recent international review of the routine immunization programme in Lao PDR recommended an increase in the number outreach services per year (WHO, 2012). Importantly, the rate of parasitic re-infection in this age-group may be high enough to result in infection shortly after antiparasitic drug intake.

In our cohort, the average height for age z-score was − 1.9, with 39 children (52.7%) showing stunting (height for age z-score < − 2). Similarly, 18 (24.3%) were underweight (weight for age < − 2 z-scores) and the prevalence of acute malnutrition, determined by weight/height or MUAC z-scores < − 2 was 2.7% and 9.5%, respectively. Although some studies have shown association of helminths with nutritional deficiencies and impaired growth, we found no statistical associations between nutritional status and presence of parasites. Furthermore, the presence of parasites was not associated with ethnicity, but was lower in infants whose mothers had a secondary education level (p = 0.04). It is clear that this study had some limitations, including the small study size (WHO recommends a sample size of above 200 in order to assess prevalence of soil-transmitted helminths (Conlan et al., 2012)), a bias in the participating groups (it is likely that the more vulnerable groups did not participate in our study) and a poor sensitivity of the formalin-ether detection technique (Glinz et al., 2010). Although this study is exploratory in nature, it can provide a useful insight into the situation in this specific context.

A study in Uganda showed that integrating deworming of pre-school children into a pre-existing child-health programme resulted in significant weight gain. These effects were greatest when treatment was bi-annual, compared to once per year (Alderman et al., 2006). Similarly, a study in Lucknow, India, showed that focusing 6-monthly rounds of single-dose deworming treatment on pre-school children resulted in significant improvements in weight gain compared to untreated children. The study authors emphasize the importance of integration into an existing infrastructure to target this age-group (Awasthi et al., 2008). Another study from Tanzania examined quarterly treatment in preschool children and found significant improvements in growth, anemia and appetite (Stoltzfus et al., 2004). Thus, there is substantial evidence of improved health following sustained treatment of pre-schoolers against helminths. In order to facilitate these treatments, integration into pre-existing public health measures such as vitamin A programmes, immunization campaigns and Child Health days may be considered. Furthermore, it is important to evaluate the effectiveness of these campaigns, monitor the safety of treatments and make dose adjustments if necessary (Albonico et al., 2008).

## 4 Conclusion

In conclusion, the high prevalence of parasitic infections among pre-school children in two districts of Huaphan province reveals incomplete coverage of the deworming programme, especially in areas remote from healthcare centres. This programme must therefore be strengthened and tightly controlled in areas of difficult access, in parallel with programmes for the supply of drinking water, sanitation and health education, such recommendations being in line with the Lao Ministry of Health guidelines on prevention and control of neglected tropical diseases (Lao Ministry of Health, 2015).Future research in Lao PDR should specify the impact of pre-school deworming on weight, anemia and reinfection rate, in order to compare different doses and schedules. Lastly, a cost-effectiveness study could confirm the need to focus antiparasitic treatment on this age group.
